# A deep learning model for generating [^18^F]FDG PET Images from early-phase [^18^F]Florbetapir and [^18^F]Flutemetamol PET images

**DOI:** 10.1007/s00259-024-06755-1

**Published:** 2024-06-11

**Authors:** Amirhossein Sanaat, Cecilia Boccalini, Gregory Mathoux, Daniela Perani, Giovanni B. Frisoni, Sven Haller, Marie-Louise Montandon, Cristelle Rodriguez, Panteleimon Giannakopoulos, Valentina Garibotto, Habib Zaidi

**Affiliations:** 1 Division of Nuclear Medicine and Molecular Imaging, Geneva University Hospital, Geneva, Switzerland; 2 Laboratory of Neuroimaging and Innovative Molecular Tracers (NIMTlab), Geneva University Neurocenter and Faculty of Medicine, University of Geneva, Geneva, Switzerland; 3 Vita-Salute San Raffaele University, Nuclear Medicine Unit San Raffaele Hospital, Milan, Italy; 4 Memory Clinic, Geneva University Hospitals, Geneva, Switzerland; 5 CIMC - Centre d’Imagerie Médicale de Cornavin, Geneva, Switzerland; 6 Faculty of Medicine, University of Geneva, Geneva, Switzerland; 7 Department of Rehabilitation and Geriatrics, Geneva University Hospitals and University of Geneva, Geneva, Switzerland; 8 Division of Institutional Measures, Medical Direction, Geneva University Hospitals, Geneva, Switzerland; 9 Department of Psychiatry, Faculty of Medicine, University of Geneva, Geneva, Switzerland; 10 CIBM Center for Biomedical Imaging, Geneva, Switzerland; 11 Department of Nuclear Medicine and Molecular Imaging, University of Groningen, Groningen, Netherlands; 12 Department of Nuclear Medicine, University of Southern Denmark, Odense, Denmark; 13 University Research and Innovation Center, Óbudabuda University, Budapest, Hungary

**Keywords:** PET, Neuroimaging, Deep learning, Metabolism, Amyloid, Deep learning

## Abstract

**Introduction:**

Amyloid-β (Aβ) plaques is a significant hallmark of Alzheimer's disease (AD), detectable via amyloid-PET imaging. The Fluorine-18-Fluorodeoxyglucose ([^18^F]FDG) PET scan tracks cerebral glucose metabolism, correlated with synaptic dysfunction and disease progression and is complementary for AD diagnosis. Dual-scan acquisitions of amyloid PET allows the possibility to use early-phase amyloid-PET as a biomarker for neurodegeneration, proven to have a good correlation to [^18^F]FDG PET. The aim of this study was to evaluate the added value of synthesizing the later from the former through deep learning (DL), aiming at reducing the number of PET scans, radiation dose, and discomfort to patients.

**Methods:**

A total of 166 subjects including cognitively unimpaired individuals (N = 72), subjects with mild cognitive impairment (N = 73) and dementia (N = 21) were included in this study. All underwent T1-weighted MRI, dual-phase amyloid PET scans using either Fluorine-18 Florbetapir ([^18^F]FBP) or Fluorine-18 Flutemetamol ([^18^F]FMM), and an [^18^F]FDG PET scan. Two transformer-based DL models called SwinUNETR were trained separately to synthesize the [^18^F]FDG from early phase [^18^F]FBP and [^18^F]FMM (eFBP/eFMM). A clinical similarity score (1: no similarity to 3: similar) was assessed to compare the imaging information obtained by synthesized [^18^F]FDG as well as eFBP/eFMM to actual [^18^F]FDG. Quantitative evaluations include region wise correlation and single-subject voxel-wise analyses in comparison with a reference [^18^F]FDG PET healthy control database. Dice coefficients were calculated to quantify the whole-brain spatial overlap between hypometabolic ([^18^F]FDG PET) and hypoperfused (eFBP/eFMM) binary maps at the single-subject level as well as between [^18^F]FDG PET and synthetic [^18^F]FDG PET hypometabolic binary maps.

**Results:**

The clinical evaluation showed that, in comparison to eFBP/eFMM (average of clinical similarity score (CSS) = 1.53), the synthetic [^18^F]FDG images are quite similar to the actual [^18^F]FDG images (average of CSS = 2.7) in terms of preserving clinically relevant uptake patterns. The single-subject voxel-wise analyses showed that at the group level, the Dice scores improved by around 13% and 5% when using the DL approach for eFBP and eFMM, respectively. The correlation analysis results indicated a relatively strong correlation between eFBP/eFMM and [^18^F]FDG (eFBP: slope = 0.77, R^2^ = 0.61, P-value < 0.0001); eFMM: slope = 0.77, R^2^ = 0.61, P-value < 0.0001). This correlation improved for synthetic [^18^F]FDG (synthetic [^18^F]FDG generated from eFBP (slope = 1.00, R^2^ = 0.68, P-value < 0.0001), eFMM (slope = 0.93, R^2^ = 0.72, P-value < 0.0001)).

**Conclusion:**

We proposed a DL model for generating the [^18^F]FDG from eFBP/eFMM PET images. This method may be used as an alternative for multiple radiotracer scanning in research and clinical settings allowing to adopt the currently validated [^18^F]FDG PET normal reference databases for data analysis.

**Supplementary Information:**

The online version contains supplementary material available at 10.1007/s00259-024-06755-1.

## Introduction

Amyloid-β (Aβ) pathology is one of the main neuropathological hallmarks of Alzheimer’s disease (AD). Aβ plaques can be detected around 10–15 years before the presence of discernible cognitive decline, which makes their detection desirable for early diagnosis of AD. The overall load and spatial distribution of brain Aβ plaques can be determined in vivo using positron emission tomography (PET), for which three fluorine-18 labelled radiotracers have been approved for clinical use [1]. In AD, the binding of Aβ tracers is widespread and notably increased in the frontal and parietal-temporal cortical regions [2]. This distribution pattern demonstrates minimal variation within individual AD patients. Strikingly, it appears to be unrelated to the clinical phenotype, the pattern of neurodegenerative changes, or the degree of cognitive decline [3]. Conversely, [^18^F]FDG-PET, the conventional radiotracer for the estimation of cerebral glucose metabolism, is well associated with synaptic dysfunction, neurodegeneration, and clinical symptoms. [^18^F]FDG-PET has value for the differential diagnosis of dementias, prediction of rapid cognitive deterioration, and staging of the extent and localization of neurodegenerative processes. For the above reasons, amyloid-PET and [^18^F]FDG-PET provide complementary information for the progression of AD-related events and clinical diagnosis as well as prognostic evaluation of patients [4].

Neural dysfunction as measured through metabolic consumption is strongly related to perfusion, which can be measured by the early-phase images of amyloid-PET suggesting their potential use as a biomarker of neurodegeneration. A dual-phase amyloid-PET acquisition protocol has been proposed, adding early phase scans to the reference late acquisition of the tracer distribution immediately after injection [5]. These early-phase images can provide a proxy for cerebral perfusion because of the high lipophilicity of the tracers. The early-phase acquisition of amyloid PET proved to have a good correlation to [^18^F]FDG PET both at group and individual levels, supporting its use as a biomarker of neuronal dysfunction [6, 7].

Despite the high overlap between hypoperfusion and hypometabolic patterns [6], the match between the two modalities is not always perfect likely because of the different biological processes involved, the noisy nature of the initial frames and the nonuniform delivery of the tracer [8].

In this regard, we aimed to assess whether the use of a deep learning (DL) model to generate synthetic [^18^F]FDG brain images from early-phase amyloid-PET brain images will ameliorate the comparability of early frames and [^18^F]FDG images using the actual [^18^F]FDG images as the gold standard.

DL has gained wide range of applications in medical imaging helping to overcome challenging tasks, such as image segmentation/classification [9], data correction (such as noise or artifact reduction) [10, 11], image interpretation (prognosis, diagnosis, and monitoring of response to treatment) [12], and cross-modality image translation or synthesis [13]. Regarding cross-modality image-to-image translation using PET imaging, DL methods have been shown to be effective in amyloid-PET to MRI image translation using generative adversarial networks (GANs) [14], and generating synthetic (R)-1-((3-([11C]methyl)pyridin-4-yl)methyl)-4-(3,4,5-trifluorophenyl)pyrrolidin-2-one ([^11^C]UCB-J) PET images from [^18^F]FDG images [15].

To the best of our knowledge, no previous studies aimed to generate [^18^F]FDG-PET images from early-phase amyloid-PET images, despite their close similarity. Hence, we proposed cross-tracer PET image translation methods using DL in order to improve the comparability between early-phase amyloid-PET images and [^18^F]FDG images obtained in cognitively unimpaired and impaired individuals from the Geneva Memory Center. Dual-phase amyloid-PET permits the assessment of neurodegeneration and Aβ pathology with a single tracer injection in one examination session, being optimal in terms of cost, patient comfort, workflow and radiation exposure.

## Material and methods

### Demographic information

The research cohort comprised patients referred to Geneva University Hospitals, spanning from cognitively unimpaired (CU) individuals to mild cognitive impairment (MCI) and dementia. Approval was obtained from the local ethics committee, ensuring adherence to the ethical principles outlined in the Declaration of Helsinki and the good clinical practice standards established by the International Conference on Harmonization. All patients signed informed consent in accordance with specific guidelines.

A total of 166 patients were included in our study and were categorized into: CU (N = 72), MCI (N = 73), and AD (N = 21) following standardized criteria for clinical staging. The inclusion criteria encompassed having at least one 3-dimensional (3D) T1-weighted MRI, undergoing dual-phase amyloid PET scans using either Fluorine-18 Florbetapir ([^18^F]FBP) (210 ± 18.77 MBq) or Fluorine-18 Flutemetamol ([^18^F]FMM) (166 ± 16.73 MBq), undergoing an [^18^F]FDG PET (203.89 ± 15.62 MBq) scan, and having an interval of less than 1 year between imaging procedures.

Table 1 presents the demographic and clinical information of our cohort. The mean time intervals between amyloid PET and [^18^F]FDG PET, between MRI and [^18^F]FDG PET, and between MRI and amyloid PET were 2.15 months (standard deviation, SD = 3.06), 1.89 months (SD = 4.15), and 2.76 months (SD = 3.40), respectively.

**Table 1:** Patient demographics of the dataset used in this study

	Whole sample	[^18^F]FBP group	[^18^F]FMM group	p-value
Number	166	94	72	
Age (mean ± SD)	73.18 ± 6.35	74.27 ± 5.548	71.76 ± 7.068	p = 0.012
gender (F/M)	98/68	58/36	40/32	p = 0.425
MMSE (mean ± SD)	25.92 ± 4.00	26.12 ± 3.857	25.66 ± 4.202	p = 0.471
Aβ status (negative/positive)	70/93	39/52	31/41	p = 0.980
Clinical Stages (CU/MCI/DEM)	72/73/21	39/40/15	33/33/6	p = 0.341

The p-values reported resulted from a t-test comparing data from the FBP and FMM subgroups.

Abbreviations: FBP = florbetapir, FMM = flutemetamol, SD = standard deviation, F = females, M = males, MMSE = Mini-Mental State Examination, Aβ = amyloid, CU = cognitively unimpaired, MCI = Mild Cognitive Impairment, DEM = dementia

As a group of comparison for the single-subject analyses, we included 112 healthy controls (HCs) who underwent [^18^F]FDG -PET and had a normal visual and semiquantitative [^18^F]FDG -PET assessment, already validated and included in previous studies [16]. We performed separate evaluations for early phase [^18^F]FBP (eFBP) and early phase [^18^F]FMM (eFMM), and the results were reported separately.

### MRI acquisition

High-resolution anatomical 3D T1 was conducted at Geneva University Hospitals’ Division of Radiology using two 3 Tesla MRI scanners (Magnetom Skyra, Siemens Healthineers, Erlangen, Germany and GE Healthcare, Milwaukee, Wisconsin) with a matrix size = 256 × 256, and 254 × 254, slice thickness = 0.9 mm and 1 mm, and repetition time = 1930 ms and 7.2 ms.

### PET acquisition

The [^18^F]FDG PET and amyloid brain PET scans were conducted at the Division of Nuclear Medicine and Molecular Imaging, Geneva University Hospitals, utilizing clinical PET scanners, including Biograph 128 mCT, Biograph 128 Vision 600 Edge, Biograph 40 mCT, or Biograph 64 TruePoint (Siemens Medical Solutions). It's important to note that all these scanners were comparable in terms of performance. The [^18^F]FDG PET scans followed the guidelines outlined by the European Association of Nuclear Medicine [17]. For amyloid PET imaging, we utilized either [^18^F]FBP (94 cases) or [^18^F]FMM (72 cases). The determination of amyloid status (Aβ + /Aβ −) for each late image was carried out by an experienced in nuclear medicine physician, following the standard operating procedures approved by the European Medicines Agency.

For the early phase amyloid PET scan (eFBP and eFMM), image acquisition commenced promptly after the injection of the tracer to obtain a static image over 5 min for eFBP and 10 min for eFMM [18, 19]. The details of the PET acquisition protocol are depicted in Supplementary Fig. 1.

### MRI and PET normalization processing

The MRI 3D T1 sequences were registered to the Montreal Neurologic Institute (MNI) space using 12 degrees of freedom using Statistical Parametric Mapping (SPM 12), which was executed within MATLAB R2018b, version 9.5 (MathWorks Inc.). The [^18^F]FDG and eFBP/eFMM images were aligned with each subject's T1 MRI and standardized to the MNI space using the transformation matrix from MRI registration. PET images underwent spatial smoothing with a 3D 8 mm Gaussian kernel. The procedures conducted were in accordance with established protocols [20].

For the quantification of Standardized Uptake Value Ratio (SUVR), we employed Automated Anatomic Labeling atlas 3 (AAL3) [21] with 166 Regions of Interest (ROIs). SUVR values were computed by standardizing the uptake within these regions against the combined mean values of the pons and cerebellar vermis, serving as the reference region. The resulting intensity-normalized PET images were saved for subsequent analyses.

### SwinUNETR model implementation

Our study introduces a novel convolution-free transformer architecture, drawing inspiration from prior works [22–24]. Our architecture features an encoder, bottleneck, decoder, and skip connections, predominantly centered around the Swin-transformer (Shifted windows) module [23].

The image processing starts by dividing input images (SUVR eFMM/eFBP images) into non-overlapping 4 × 4 blocks, linearly projecting them to create sequences for network input. The encoder utilizes patch-merging blocks for down-sampling and Swin-transformer blocks for representation learning, forming a hierarchical structure akin to the U-Net's architecture. The symmetric decoder employs Swin-transformer layers and patch-expanding units.

To facilitate signal transmission, skip connections are established between the encoder and decoder. At the encoder's core, a bottleneck comprises two consecutive Swin-transformer blocks, serving as an additional connection between the encoder and decoder without involving up- or down-sampling operations.

**Fig. 1:**
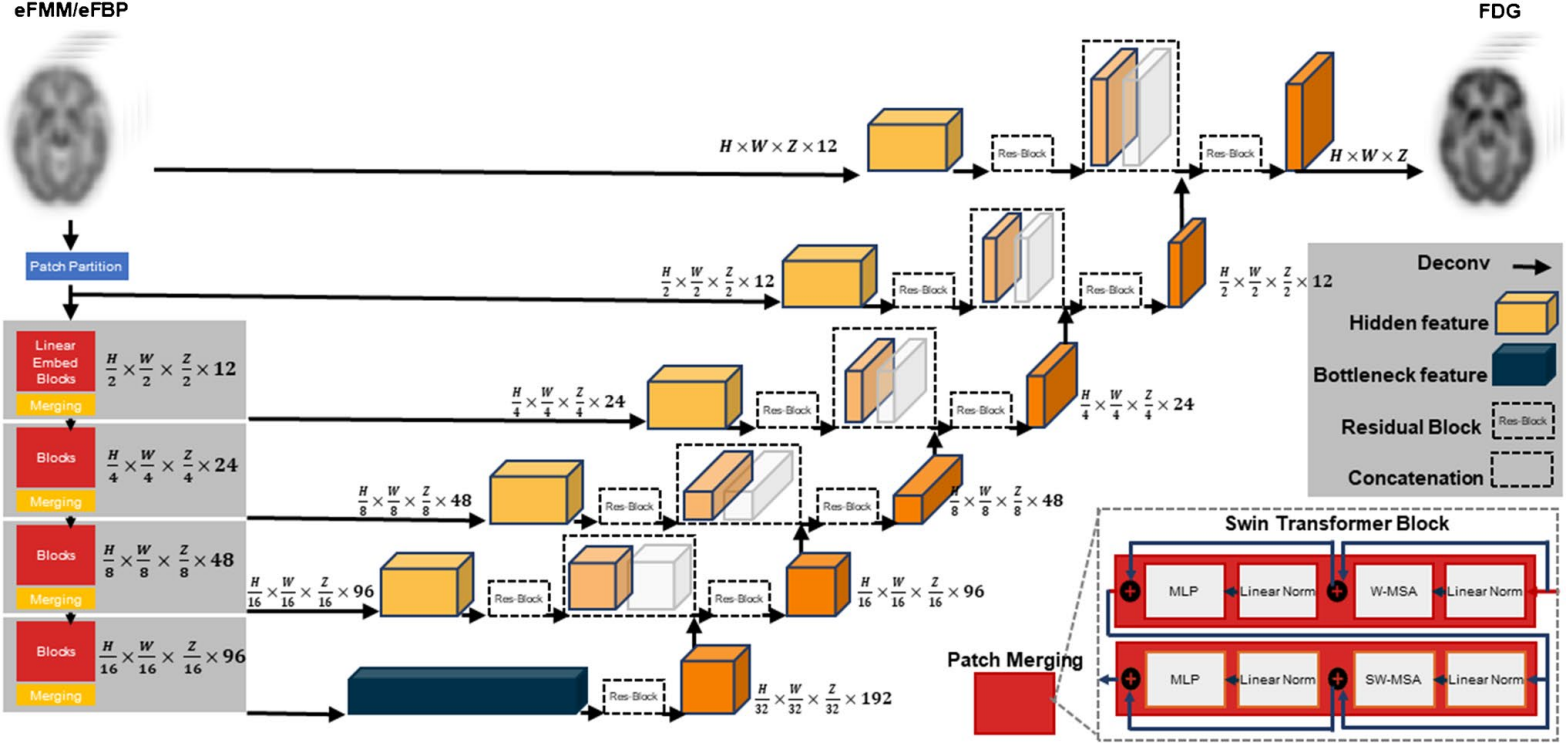
Overview of the Swin UNETR architecture. The input to our model is a single early phase eFBB/eFMM images (different models for eFBP/eFMM were trained separately) and the output is the synthetic [^18^F]FDG. The Swin UNETR creates non-overlapping patches of the input data and uses a patch partition layer to create windows with a desired size for computing the self-attention. The encoded feature representations in the Swin transformer are fed to a CNN-decoder via skip connections at multiple resolutions

The Swin-transformer block, inspired by shifted-windows [23], employs patch division at one level and a shifted version at the next, enabling connections between different window shapes via self-attention mechanisms. Comprising layer normalization (LN), multi-head self-attention (MSA), multi-layer perceptrons (MLP), and multiple skip-connections, this block ensures efficient information flow. The architecture of SwinUNETR is illustrated in Fig. 1.

The mean square error (MSE) loss function was employed as the guiding loss function for our model, which underwent training via a five-fold cross-validation methodology (60%, 20% and 20% allocation for the training, validation, and testing, respectively). The images were maintained within the SUVR range without undergoing any normalization procedure. Training the model extended over 300 epochs, concluding upon reaching a plateau in the graph representing the loss function.

### Assessment of image quality

Initially, the predicted images underwent a visual inspection to detect potential artifacts and abnormalities, with a subsequent effort to identify the underlying causes and provide detailed reports for documentation. In our research, the evaluation of the DL model's performance was conducted by assessing various metrics, including the Structural Similarity Index (SSIM), Root Mean Squared Error (RMSE), and Peak Signal-to-Noise Ratio (PSNR). These metrics were computed for early phase amyloid image and synthetic [^18^F]FDG while considering the actual [^18^F]FDG scan as the ground truth. Subsequently, the metrics for each section were averaged to derive a comprehensive assessment. A pairwise t-test was computed individually across all groups, employing a predetermined significance level of 0.05. The distributions of SUVR for all regions and patients were visualized by plotting a Bland–Altman graph for both eFBP/eFMM and [^18^F]FDG, and synthetic [^18^F]FDG and [^18^F]FDG.

### Clinical evaluation

To evaluate the performance of our model in clinical setting, two experienced nuclear medicine physicians (V.G with 18 years and G.M with 5 years’ experience in nuclear medicine and reading of brain PET scans) evaluated and compared the physiological aspects and biodistribution patterns of [^18^F]FDG images (as standard of reference) and synthetic [^18^F]FDG and eFBP/eFMM. We hypothesized that if the model enhances the similarity between [^18^F]FDG images and synthetic [^18^F]FDG compared to eFBP/eFMM images, our model can improve the accuracy of clinical diagnosis. To evaluate this hypothesis, 30 subjects were selected randomly and the synthetic [^18^F]FDG and eFBP/eFMM were anonymized while keeping the [^18^F]FDG known. We asked the physicians to look at the images head-to-head (actual [^18^F]FDG beside image unknown-1 and image unknown-2) and select a clinical similarity score (CSS) between 1 to 3 when comparing unknown images with actual [^18^F]FDG images. The scores were selected as follows:

1. *No clinical similarity:* The unknown image compared to actual [^18^F]FDG does not represent similar clinical information.
2. *Slightly similar:* The unknown image compared to actual]^18^F]FDG leads to partially similar diagnosis, some important information was missed.
3. Similar: The unknown image compared to actual [^18^F]FDG leads to similar diagnosis, the necessary information was preserved.

An Intraclass Correlation Coefficient (ICC) was calculated between the two physicians to measure the agreement and consistency between assigned ranks.

### Single-subject voxel-wise analyses

According to a validated SPM-based single-subject procedure [16], each PET and synthetic PET image was tested for relative hypometabolism/hypoperfusion by means of a 2-sample t-test in comparison with [^18^F]FDG PET images of 112 HC subjects. The statistical threshold for the resulting hypometabolic and hypoperfusion SPM maps was set at a P-value of 0.05, uncorrected for multiple comparisons, considering significant clusters containing more than 100 voxels. SPM maps were then binarized for further Dice coefficient analyses.

### Statistical analysis

Dice coefficients were calculated using FSL software [25] to quantify the whole-brain spatial overlap between hypometabolic ([^18^F]FDG PET) and hypoperfused (eFBP/eFMM) binary maps at the single-subject level as well as between [^18^F]FDG PET and synthetic [^18^F]FDG PET hypometabolic binary maps. Dice coefficient for binary maps A and B is defined as: *Dice* = 2 ∗ (*A* ∩ *B*) /(*A* + *B*). It takes the value of 1 if *A* and *B* assume the same logical value in every pixel (high concordance), and a value of 0 if they always disagree (null concordance). It is interpreted as follows: < 0.2, poor; 0.2–0.4, fair; 0.4–0.6, moderate; 0.6–0.8, good; and > 0.8, excellent agreement.

General linear models were performed to assess the correlation between eFBP/eFMM SUVR in the AAL ROIs and their respective [^18^F]FDG SUVR as well as between [^18^F]FDG SUVR and their respective synthetic [^18^F]FDG SUVR in the whole sample. To evaluate the level of statistical significance between two groups, namely eFBP/eFMM vs. reference [^18^F]FDG and synthetic [^18^F]FDG vs. reference [^18^F]FDG, we performed a paired samples t-test. A P-value less than 0.05 was used as threshold for statistical significance.

## Results

### Qualitative and quantitative assessment

**Fig. 2:**
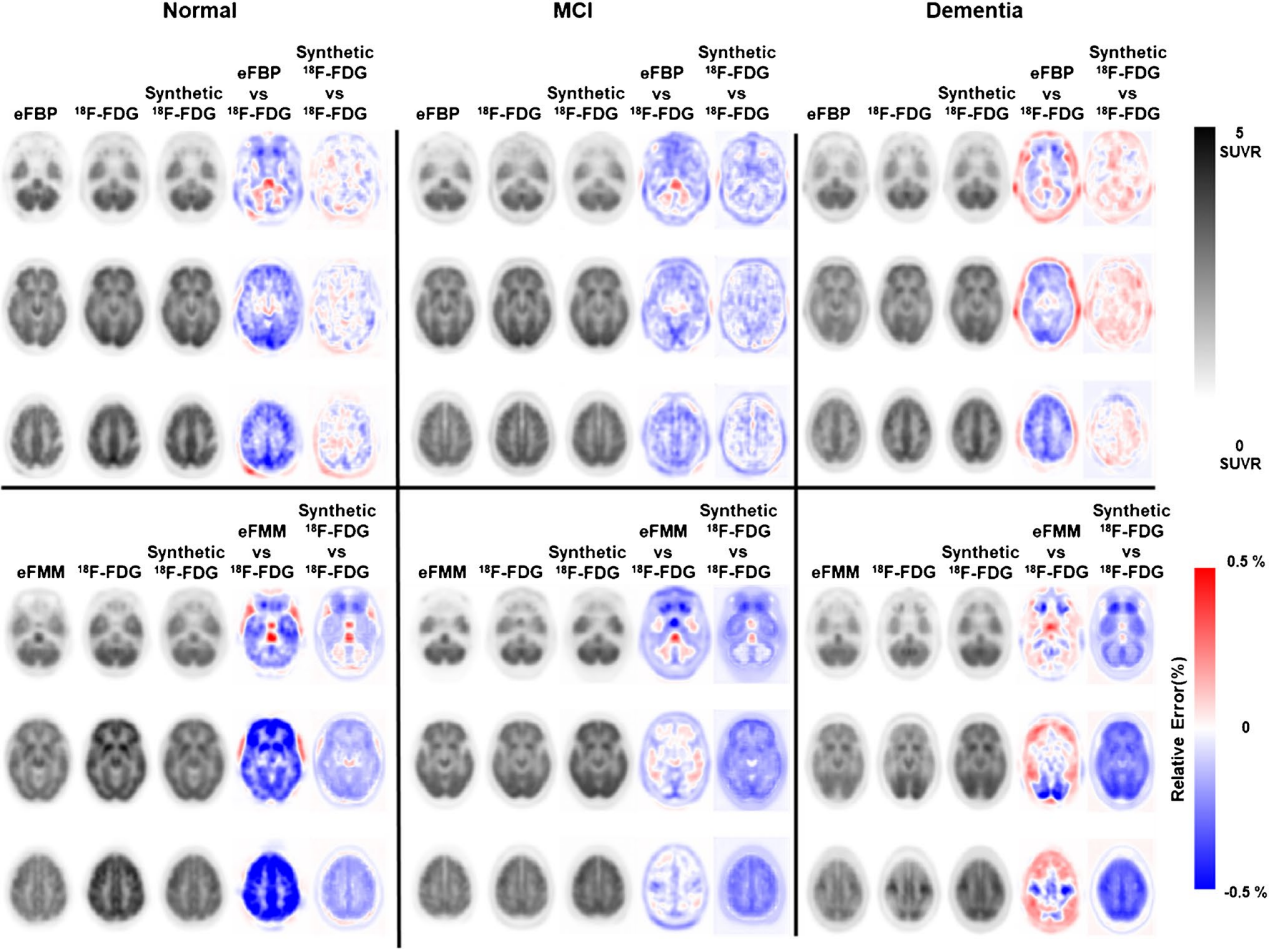
Six different subjects with various clinical status (Normal, MCI, Dementia), for eFBP and eFMM. The standard of reference eFBP/eFMM and [^18^F]FDG are shown in the first and second column. The generated synthetic [^18^F]FDG from eFBP/eFMM is shown in the third column. The difference map between the reference eFBP/eFMM and [^18^F]FDG and generated synthetic ^18^F-FDG vs reference [^18^F]FDG are shown in the last two columns. The images range is between 0 to 5 SUVR and the range of the difference map is between -0.5 to + 0.5 SUVR

The initial visual assessment between the standard of reference [^18^F]FDG, eFBP/eFMM, and synthetic [^18^F]FDG illustrates the performance of the proposed model for generating synthetic [^18^F]FDG, which are in good agreement with the actual [^18^F]FDG (Fig. 2). This visual inspection is supported by the difference maps calculated by subtracting the eFBP/eFMM and synthetic [^18^F]FDG images from the actual ^18^F-FDG images. The difference maps before and after applying the DL model show less and uniform bias. The visual assessment is supported by quantitative metrics, such as PSNR, SSIM, and RMSE, listed in Table 2. The results demonstrate that there is RMSE reduction to around 40% and 31% for eFBP and eFMM, respectively, after applying the DL model for generating synthetic ^18^F-FDG. The SSIM improved from 0.91 ± 0.02 to 0.94 ± 0.04 and from 0.88 ± 0.03 to 0.90 ± 0.05 for eFBP and eFMM, respectively. The p-value for all metrics and all paired groups (eFBP/eFMM vs. [^18^F]FDG and synthetic [^18^F]FDG vs. [^18^F]FDG) was lower than 0.05 reflecting statistical significance of the differences.

**Table 2:** Quantitative metrics for [^18^F]FBP, [^18^F]FMM. The Structural Similarity Index (SSIM), Root Mean Squared Error (RMSE), and Peak Signal-to-Noise Ratio (PSNR) were calculated before ([^18^F]FDG vs. early phase amyloid) and after ([^18^F]FDG vs. synthetic [^18^F]FDG) using deep learning methods to demonstrate the performance of deep learning in improving the similarity between synthetic [^18^F]FDG and actual [^18^F]FDG. Higher SSIM (close to 1) show higher structural similarity and higher PSNR, whereas lower RMSE represent lower noise and error, respectively

Quantitative metrics		
Metric	**eFBP**	**eFMM**
SSIM: Synthetic ^18^F-FDG vs. [^18^F]FDG	0.94 ± 0.04	0.90 ± 0.05
SSIM: Early phase amyloid vs. [^18^F]FDG	0.91 ± 0.02	0.88 ± 0.03
RMSE: Synthetic [^18^F]FDG (SUVR) vs. [^18^F]FDG	0.06 ± 0.03	0.06 ± 0.02
RMSE: Early phase amyloid (SUVR) vs. [^18^F]FDG	0.11 ± 0.02	0.11 ± 0.02
PSNR: Synthetic [^18^F]FDG (dB) vs. [^18^F]FDG	30.15 ± 2.19	30.60 ± 2.92
PSNR: Early phase amyloid (dB) vs. [^18^F]FDG	25.42 ± 1.70	25.92 ± 1.90

### Clinical evaluation

**Fig. 3:**
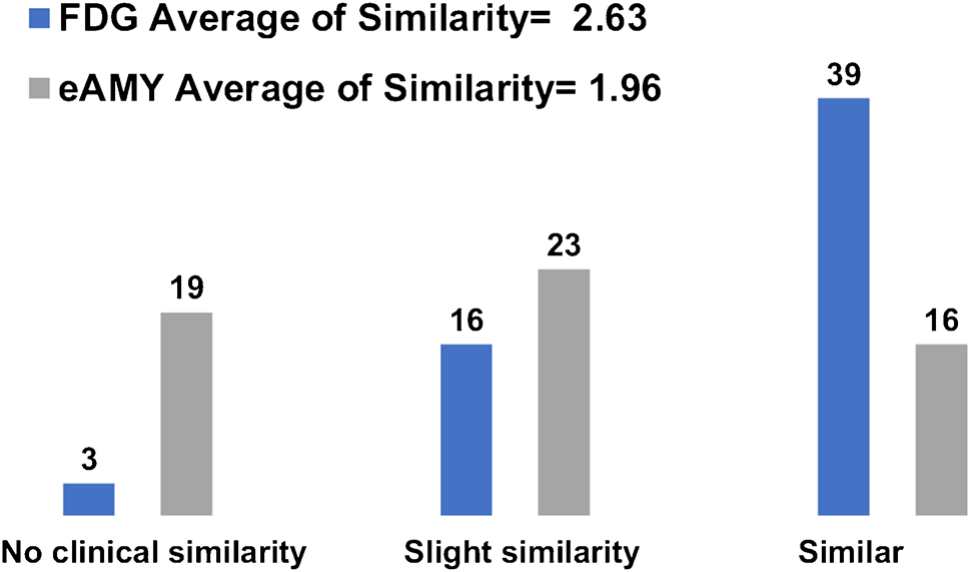
Clinical evaluation of the generated synthetic [^18^F]FDG versus eFMM/eFBP images showing the average scores assigned by two experienced nuclear medicine physicians. The synthetic [^18^F]FDG and eFMM/eFBP images were compared with the corresponding standard of reference [^18^F]FDG images and a similarity score between 1–3 assigned. Score 1 means there is not any similar clinical information between the synthetic [^18^F]FDG or eFMM/eFBP images and reference [^18^F]FDG image, score 2 means there is slightly similar information and score 3 means they provide similar clinical information, and may lead to similar clinical diagnosis

The clinical assessment showed that the synthetic [^18^F]FDG images are more similar to the actual [^18^F]FDG images in terms of preserving clinically relevant uptake patterns in comparison to eFBP/eFMM (Fig. 3). The average of clinical image similarity score for eFBP/eFMM was 1.96 and increased to 2.63 for synthetic [^18^F]FDG. Prior to applying the proposed model, there were 19 eFBP/eFMM subjects with no clinical image similarity (score 1) with [^18^F]FDG. This reduced to 3 subjects after applying the DL model. Figure 3 also shows that there were only 16 eFBP/eFMM subjects with similar clinical patterns (score 3) with actual [^18^F]FDG, while this increased to 39 subjects after applying the DL model. Alongside the images, clinical information provided includes Mini-Mental State Examination (MMSE), age, sex and amyloid status.

### Correlations between eFBP/eFMM and [^18^F]FDG SUVR

**Fig. 4:**
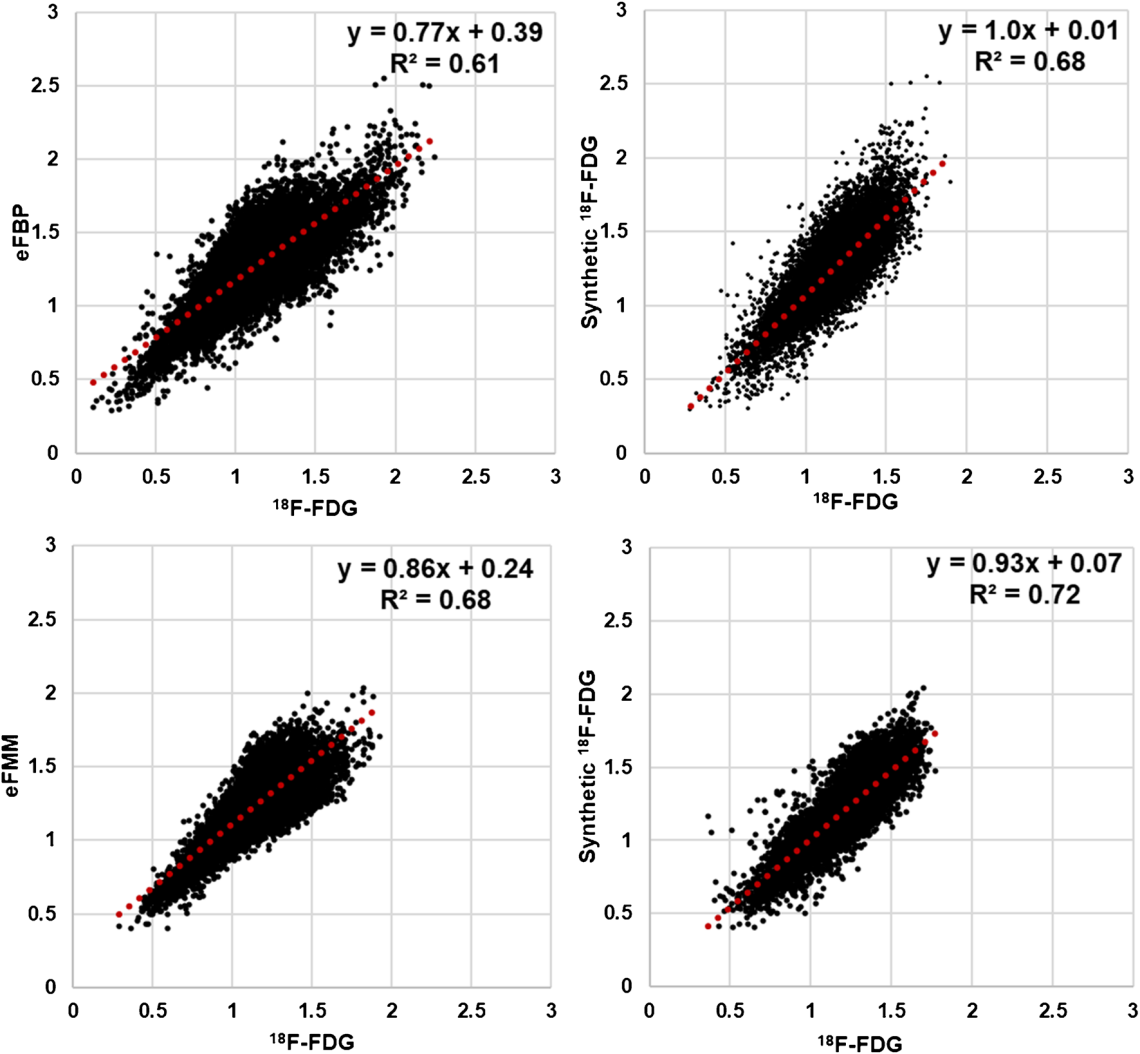
Region-wise correlation between early phase amyloid images (eFBP and eFMM) and [^18^F]FDG images. Each black point represents the activity of a region for a subject. 166 regions for each eFBP/eFMM subjects were calculated and reported. The unit is SUVR

**Fig. 5:**
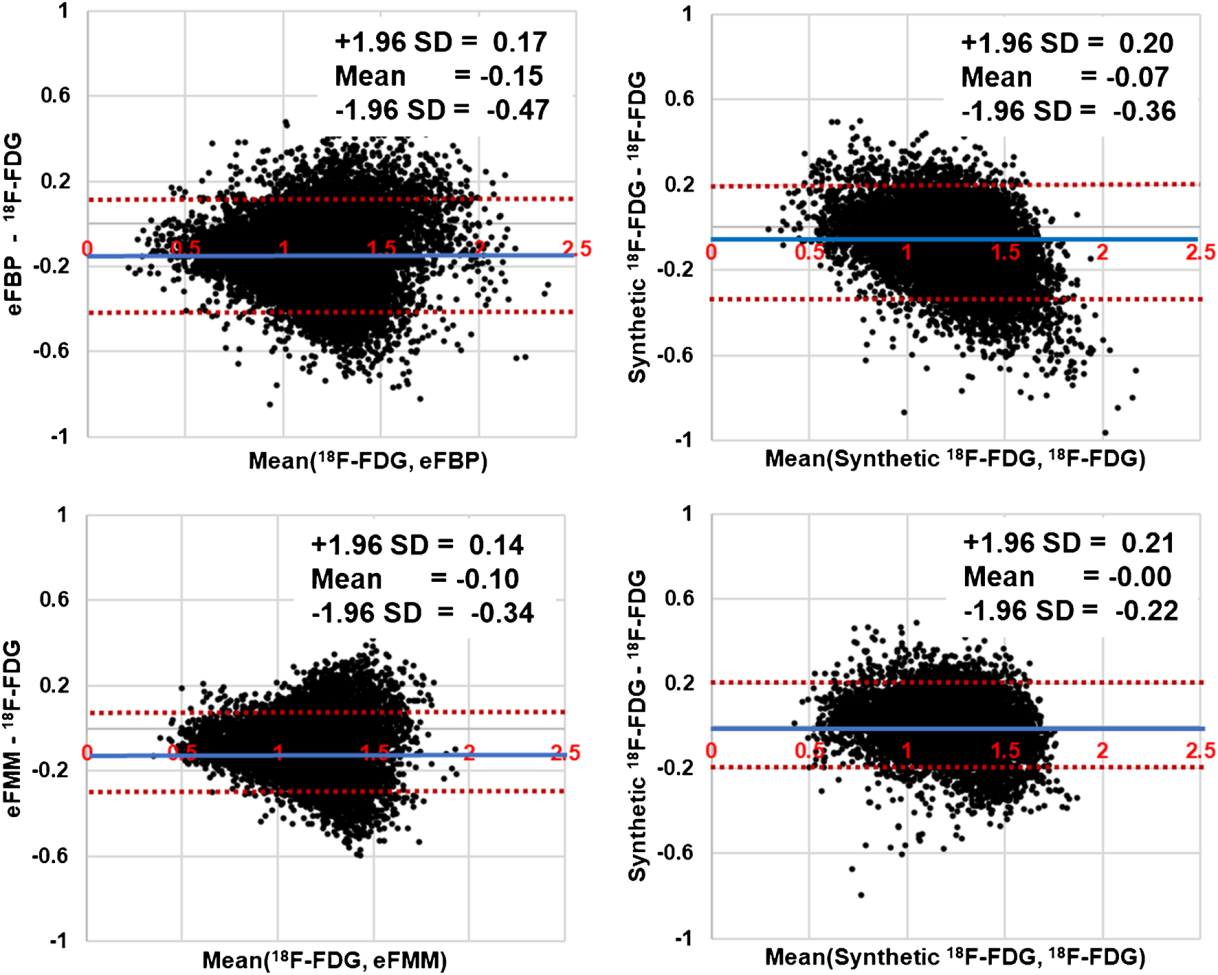
Bland & Altman analysis. Each black point represents the activity of a region for a subject. 166 regions for each eFBP, eFMM subject were calculated and reported. The unit is SUVR. The blue line represents the mean, whereas the red dashed lines represent the upper and lower confidence interval

The first column of Fig. 4 represents the region-wise correlation between eFBP/eFMM and [^18^F]FDG. Each black point indicates the SUVR in 166 regions and 166 patients. The results show a relatively strong correlation between eFBP/eFMM and [^18^F]FDG (eFBP: slope = 0.77, R^2^ = 0.61, P-value < 0.0001; eFMM: slope = 0.77, R^2^ = 0.61, P-value < 0.0001). These correlations were significantly improved when eFBP/eFMM were used as inputs of our DL model and the synthetic [^18^F]FDG were generated (synthetic [^18^F]FDG generated from eFBP (slope = 1.00, R^2^ = 0.68, P-value < 0.0001), eFMM (slope = 0.93, R^2^ = 0.72, P-value < 0.0001)). The Bland & Altman analysis is depicted in Fig. 5, where each black point indicates the region SUVR for all the subjects. The results show that applying our DL model, the mean of bias between all regions reduced from -0.15 and -0.10 SUVR to -0.07 and 0.00 SUVR for eFBP and eFMM, respectively. Table 3 presents the average SUVR bias for all regions and all subjects before and after using the DL model. The average was reported separately for amyloid-positive and negative subjects to evaluate the performance of the model for different amyloid status. The average error for all amyloid positive cases reduced from -10.31 ± -7.20% and -10.63 ± -1.95% to -0.91 ± 0.45% and -5.32 ± 6.60% for [^18^F]FBP and [^18^F]FMM, respectively. The confidence interval range reduced from 0.64 and 0.48 to 0.56 and 0.43 for eFBP and eFMM, respectively (P-value < 0.0001).

**Table 3:** Subject-wise error analysis. Average error for all regions between all [^18^F]FBP and [^18^F]FMM subjects. The average of errors for subjects with positive and negative amyloid status were also reported separately

		eFBP vs [^18^F]FDG for all regions		Synthetic [^18^F]FDG vs [^18^F]FDG for all regions	
Diagnosis	Amyloid status	Mean	SD	Mean	SD
Dementia	Positive	-7.74	1.58	2.05	6.11
Dementia	Negative	-10.27	0.00	-4.08	0.00
MCI	Positive	-6.68	-6.68	2.27	2.27
MCI	Negative	-7.40	6.69	-0.21	6.69
Control	Positive	-16.49	-16.49	-7.04	-7.04
Control	Negative	-7.85	3.38	1.59	4.10
All	Positive	-10.31	-7.20	-0.91	0.45
All	Negative	-8.51	3.36	-0.90	3.59
		eFMM vs [^18^F]FDG for all regions		Synthetic [^18^F]FDG vs [^18^F]FDG for all regions	
Diagnosis	Amyloid status	Mean	SD	Mean	SD
Dementia	Positive	-9.44	4.70	-4.19	9.62
Dementia	Negative	-7.51	8.72	-5.66	5.00
MCI	Positive	-8.64	3.26	-4.77	4.72
MCI	Negative	-9.23	5.86	-5.19	7.65
Control	Positive	-13.80	-13.80	-7.00	5.44
Control	Negative	-15.77	-15.77	-4.89	5.60
All	Positive	-10.63	-1.95	-5.32	6.60
All	Negative	-10.84	-0.40	-5.25	6.08

### Single-subject eFBP/eFMM and [^18^F]FDG patterns

**Fig. 6:**
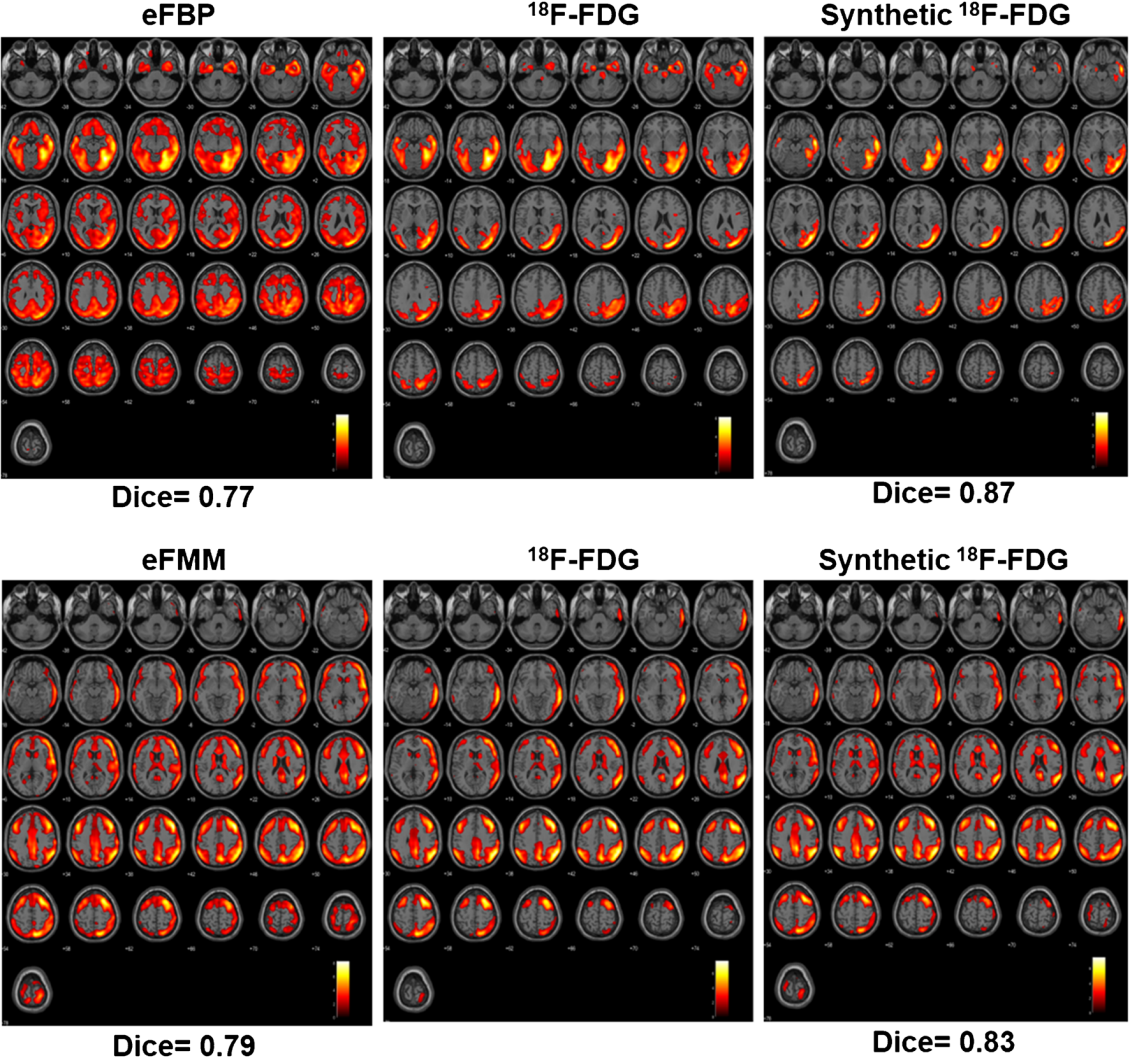
The SPM-t maps of hypoperfusion/hypometabolism of two single cases of AD patients for the two tracers (eFMM/eFBP), shown as example. All t-maps for each case were produced compared to the same set of [^18^F]FDG PET scans in the healthy control subjects. Yellow/red scales shown in SPM maps are regions which are hypoperfused/hypometabolic in these patients in comparison to the normal control database (see text for details)

Figure 6 shows representative examples of single-subject results of statistical difference between the healthy control [^18^F]FDG used as reference and AD patients for eFBP, eFMM,[^18^F]FDG and synthetic [^18^F]FDG (Fig. 6). The map shows voxels with significant differences in comparison with reference healthy control [^18^F]FDG. The middle column can be considered as the ground truth activity concentration, whereas the first and last columns are the maps belonging to eFBP/eFMM and synthetic [^18^F]FDG. The synthetic [^18^F]FDG shows more overlap with the ground truth as reflected by the higher Dice score. At the group level, the Dice scores improved by around 13% and 5% when using our DL approach for eFBP and eFMM, respectively. Table 4 shows the Dice coefficients indicating the voxel-by-voxel concordance between synthetic [^18^F]FDG and [^18^F]FDG maps and between eFBP/eFMM and [^18^F]FDG maps. The average Dice score improved significantly (P-value < 0.05) from 0.51 to 0.6 for eFBP and from 0.54 to 0.56 (P-value = 0.24) for eFMM samples, respectively.

**Table 4:** Dice results. The single-subject procedure consists in voxel-wise t-test with a large dataset of [^18^F]FDG scans of healthy controls in order to obtain hypoperfusion and hypometabolism maps. The Dice coefficient was calculated to investigate voxel-by-voxel concordance/overlap between the hyperfusion/hypometabolism maps obtained with different modalities, namely [^18^F]FDG/eFBP/eFMM and synthetic [^18^F]FDG. Here, the Dice coefficients indicating the voxel-by-voxel concordance between synthetic [^18^F]FDG and [^18^F]FDG maps and between eFBP/eFMM and [^18^F]FDG maps

eFBP	eFBP	Synthetic [^18^F]FDG	p-value
Average of Dice	0.51	0.60	< 0.05
Standard deviation	0.19	0.16	
eFMM	eFMM	Synthetic [^18^F]FDG	
Average of Dice	0.54	0.56	0.24
Standard deviation	0.15	0.18	

**Fig. 7:**
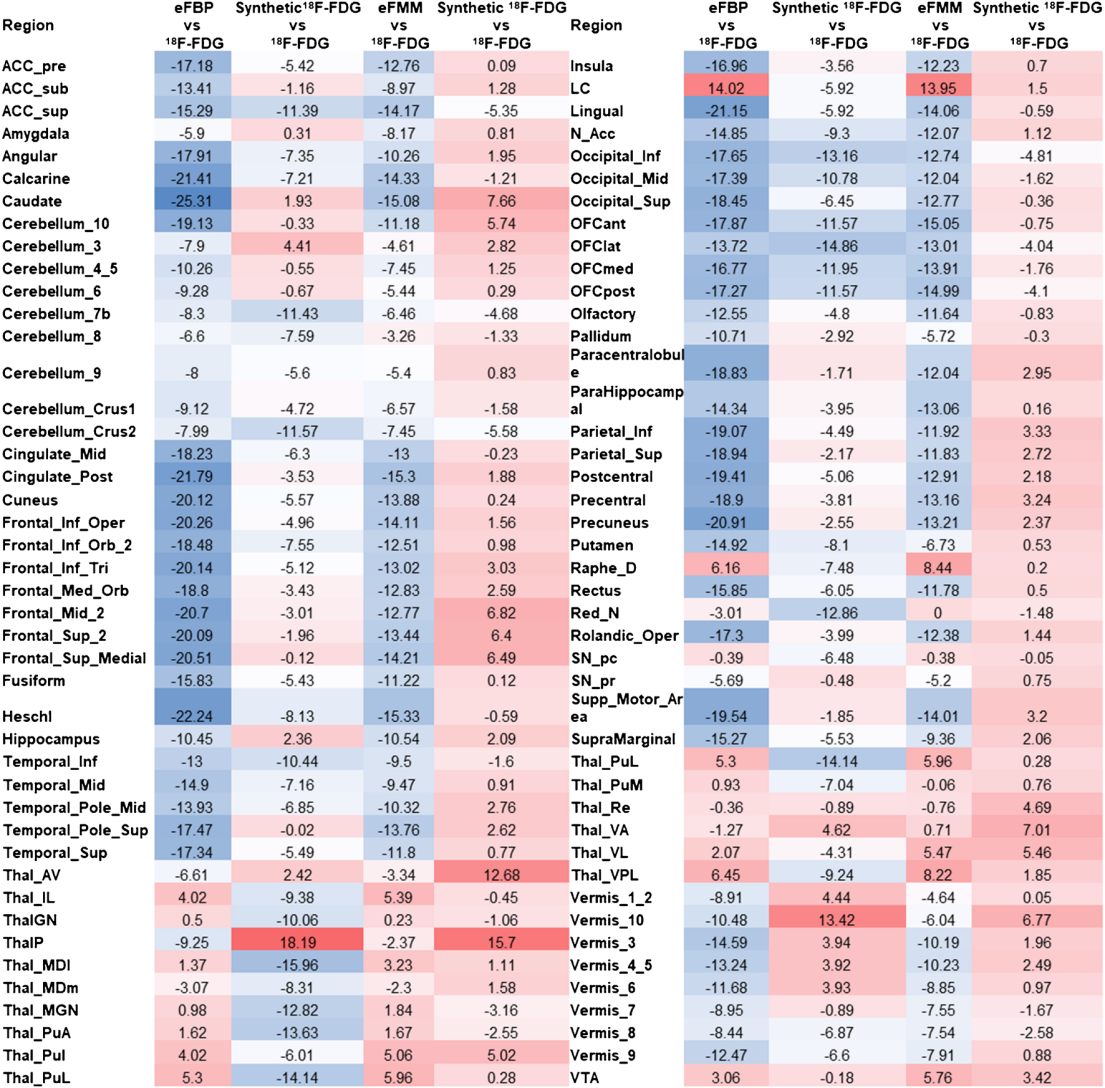
Region-wise error analysis. Average error for each AAL region between all the eFBP and eFMM subjects. The average of left and right side was reported to simplify the presentation

Figure 7 shows the heatmap for the average of absolute errors for each region between eFBP/eFMM and [^18^F]FDG and synthetic [^18^F]FDG and [^18^F]FDG. The average of left and right sides was reported for simpler representation. Of the 88 regions (average of left and right), there were only 23 and 10 regions with lower bias in eFBP and eFMM, respectively, while the rest of the regions had a lower bias in synthetic [^18^F]FDG.

## Discussion

This study introduced a DL model to generate [^18^F]FDG brain PET images from early-phase Amyloid PET images, with the aim to integrate imaging modalities and streamline clinical practice. The developed model may combine two imaging modalities, allowing [^18^F]FDG brain PET images to be extrapolated from existing early-phase Amyloid PET data. This integration significantly reduced the scanning time, cost and radiation dose typically required for separate scans. In each single PET scan, an individual may receive an effective dose around 3.8, 5.6, and 3.9 mSv for [^18^F]FDG, [^18^F]FMM, and [^18^F]FBP, respectively [26–28], which might increase in the case of follow-up scans PET scanning could be challenging for dementia patients owing to the potential occurrence of motion artifacts. Hence, methods that can reduce the scanning time and injected activity is of high clinical relevance [29–31].

In a recent work, Wang et al. used a 3D U-NET model to generate [^11^C]UCB-J PET images of synaptic vesicle protein 2A (SV2A), a substitute of synaptic density, and ^11^C-PiB directly from [^18^F]FDG PET [15]. They reported a reasonable prediction accuracy under 10% for ROI-based bias estimation. The driving force of our work is a solid documentation of the correlation between the early phase amyloid and [^18^F]FDG reported in previous studies [6, 32]. The high lipophilicity of amyloid tracers makes early-phase images a useful proxy for brain perfusion [5, 33], which is strictly correlated with neuronal dysfunction as determined by metabolic consumption [34, 35]. Based on neurovascular coupling, the relationship between cerebral perfusion and metabolism in aging and dementia diseases has long been known. Early-phase amyloid PET has demonstrated a strong association with [^18^F]FDG PET uptake, indicating its potential application as a surrogate measure of neurodegeneration in AD [6, 7]. When comparing eFBP/eFMM single-subject analysis to controls, significant hypoperfusion clusters emerged in the presence of neurodegeneration as determined by [^18^F]FDG PET. In line with our previous study [6], these clusters demonstrated a good correlation with the brain hypometabolism topography. Although we showed the possibility of using DL models for generating synthetic [^18^F]FDG PET images from early phase amyloid images, at this stage we cannot claim that the generated images can replace actual [^18^F]FDG images. However, these synthetic [^18^F]FDG images can be useful in research setting or monitoring purpose as an additional information for diagnosis, especially when the [^18^F]FDG PET scan is not available or multiple scans might lead to radiation hazards to the subject. Our work demonstrated significant positive correlations between synthetic [^18^F]FDG and [^18^F]FDG SUVR in a memory clinic sample, which was even higher than the correlations found between eFMM/eFBP SUVR and [^18^F]FDG SUVR. The association did not depend on the amyloid tracers that were utilized to generate synthetic images. Moreover, clusters of significant hypometabolism were found in patients compared to controls when we performed the SPM single-subject analysis on synthetic [^18^F]FDG pictures. These clusters showed a good correlation to the original hypometabolism maps, and the Dice scores were higher for synthetic [^18^F]FDG than eFMM/eFBP, indicating higher topographical agreement with [^18^F]FDG.

Our DL models performed slightly better for eFBP compared to eFMM. Our hypothesis is that this might be due to the amount of injected activity for eFBP (~ 210 MBq), which is higher than eFMM (~ 166 MBq). The higher injected activity leads to a stronger signal and hence higher signal-to-noise ratio (SNR) and overall better image quality. Conversely, the SSIM metric indicated that the structural similarity is higher between eFBP and [^18^F]FDG (0.91 ± 0.02) compared to eFMM and [^18^F]FDG (0.88 ± 0.03). As a result, the generated [^18^F]FDG images from eFBP appear more similar to [^18^F]FDG images, than those generated from eFMM.

The assessment of our model confirmed the overall good performance, yet it also highlighted substantial inconsistency between readers, as denoted by an ICC close to zero. Notably, the two readers exhibited differing observations regarding the similarity of eAMY cases with [^18^F]FDG. Despite this discrepancy, both reviewers reported a consistent trend. This incongruity underscores the subjective nature of visual rating, emphasizing the necessity to acknowledge and address biases inherent in such evaluations. Moving forward, implementing measures to standardize the assessment process and explore alternative analytical approaches can enhance the reliability and objectivity of our model evaluation, ensuring more robust and valid conclusions. The limited sample size is another limitation of the present work.

Apart from the evidence of a correlation between early-phase amyloid and [^18^F]FDG, it is worth mentioning that the differences in biological information obtained from eFMM/eFBP and [^18^F]FDG tracers might influence diagnostic sensitivity and specificity, potentially affecting clinical interpretation. Although our clinical evaluation demonstrated that the synthetic [^18^F]FDG images generated by our model have higher clinical similarity to actual [^18^F]FDG images compared to eFMM/eFBP, exploiting the biological framework remains a subject of consideration. The clinical implications of fusing Amyloid and [^18^F]FDG PET imaging are substantial, offering comprehensive brain assessment for ongoing patient monitoring.

## Conclusion

The proposed DL model may represent an advancement in eFMM/eFBP and [^18^F]FDG PET imaging. While offering potential benefits in efficiency and cost-effectiveness, further validation and refinement are essential. Addressing the biological variability and ensuring diagnostic accuracy are pivotal in establishing the clinical utility of this integrated imaging approach.

## Supplementary Information

Below is the link to the electronic supplementary material.

Supplementary file1 (PDF 171 KB)

## Data Availability

The data used in this work is not available.
