# Supplementary material for: A deep learning model for generating [18F]FDG PET Images from early-phase [18F]Florbetapir and [18F]Flutemetamol PET images
Source: Eur J Nucl Med Mol Imaging. 2024 Jun 11;51(12):3518–31. doi: 10.1007/s00259-024-06755-1 (PMC11445334; doi:10.1007/s00259-024-06755-1)
Supplement: Supplementary file 1 — Supplementary file1 (PDF 171 KB) [file 259_2024_6755_MOESM1_ESM.pdf]

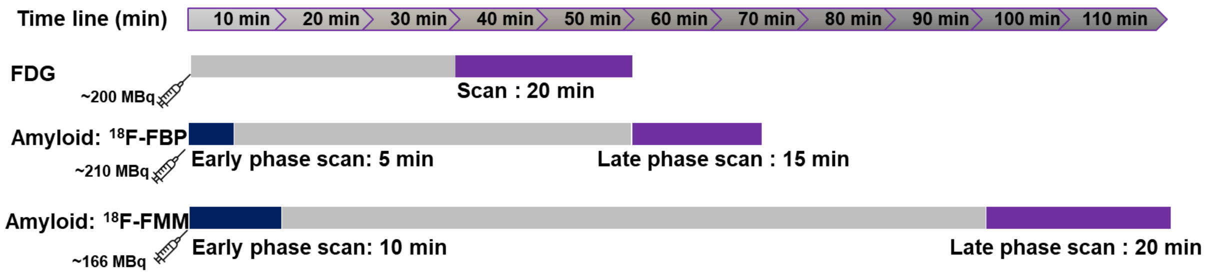

Supplementary Figure 1: The PET acquisition protocol for ^{18}F-FDG, eFBP and eFMM.

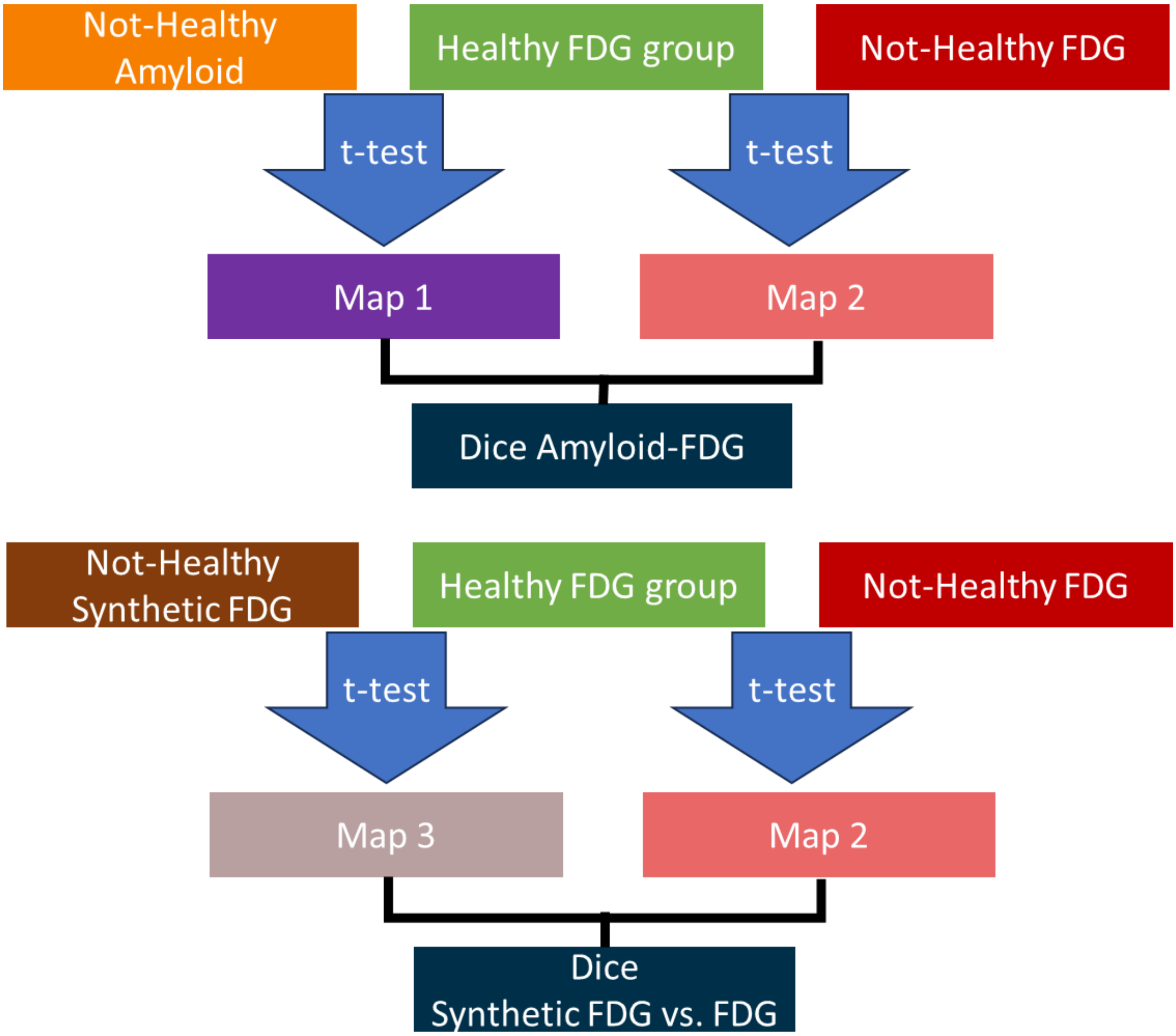

Supplementary Figure 2S. Dice score calculation strategy.
